# Keystone Veterinary Medical Association

**Published:** 1895-09

**Authors:** W. L. Rhoads

**Affiliations:** Secretary


### KEYSTONE VETERINARY MEDICAL ASSOCIATION.

THE last meeting for the session of 1894–95 was held at the Runnymede Club, Lansdowne, June 11, 1895, by invitation of Dr. Rhoads, and was well attended.

In the absence of President Lintz, Dr. Bridge presided. After the roll-call and reading of the minutes of the May meeting, Dr. U. S. G. Beeber, of Kutztown, was elected to membership.

Dr. John R. Hart was appointed to draft a letter to the Governor regarding appointments on State Veterinary Medical Examining Boards.

This being the last meeting of the current year, the regular routine of business and literary exercises was dispensed with, and the evening devoted to general business topics and social enjoyment.

Those present were U. S. G. Bieber, J. L. Bradley, F. Bridge, A. O. Cawley, C. T. Goentner, W. H. Hoskins, J. D. Houldsworth, H. J. McClellan, B. F. Sensemen, and W. L. Rhoads.

After a very pleasant evening the meeting adjourned, to meet at the office of Dr. W. H. Hoskins, September 17, 1895.

W. L. RHOADS,
Secretary.

[The Association will resume its regular monthly meetings on September 17th. Reports of her delegates at Cresson Springs, on the 3d, and at Des Moines, Iowa, on the 10th, 11th, and 12th, will be received; the winter's work will be planned, and matters of much importance to the welfare of the profession will receive consideration.]

